# Standard reference values of the postural control in healthy female adults aged between 31 and 40 years in Germany: an observational study

**DOI:** 10.1186/s40101-020-00229-7

**Published:** 2020-09-09

**Authors:** Daniela Ohlendorf, Julia Pflaum, Christina Wischnewski, Sebastian Schamberger, Christina Erbe, Eileen M. Wanke, Fabian Holzgreve, David A. Groneberg

**Affiliations:** 1 Institute of Occupational Medicine, Social Medicine and Environmental Medicine, Goethe University Frankfurt, Theodor-Stern-Kai 7, Building 9a, 60596 Frankfurt, Germany; 2 School of Dentistry, Department of Orthodontics, Goethe University Frankfurt, Frankfurt, Germany; 3 University Medical Center, Department for Orthodontics, Johannes Gutenberg University Mainz, Mainz, Germany

**Keywords:** Postural control, Female subjects, Standard value

## Abstract

**Background:**

To detect deviations from a normal postural control, standard values can be helpful for comparison purposes. Since the postural control is influenced by gender and age, the aim of the present study was the collection of standard values for women between 31 and 40 years of age.

**Methods:**

For the study, 106 female, subjectively healthy, German subjects aged between 31 and 40 years (35 ± 2.98 years) were measured using a pressure measuring platform.

**Results:**

Their average BMI was 21.60 ± 4.65 kg/m^2^. The load distribution between left and right foot was almost evenly balanced with a median 51.46% load on the left [tolerance interval (TR) 37.02%/65.90%; confidence interval (CI) 50.06/52.85%] and 48.54% [TR 43.10/62.97%; CI 47.14/49.93%] on the right foot. The median forefoot load was 33.84% [TR 20.68/54.73%; CI 31.67/37.33%] and the rearfoot load was measured at 66.16% [TR 45.27/79.33%; CI 62.67/68.33%]. The median/mean body sway in the sagittal plane was measured 12 mm [TR 5.45/23.44 mm; CI 11.00/14.00 mm] and 8.17 mm in the frontal plane [TR 3.33/19.08 mm; CI 7.67/9.33 mm]. The median of the ellipse area is 0.72 cm^2^ [TR 0.15/3.69 cm^2^; CI 0.54/0.89°]. The ellipse width has a median of 0.66 cm [TR 0.30/1.77 cm; CI 0.61/0.78 cm] and the height of 0.33 cm [TR 0.13/0.71 cm; CI 0.30/0.37 cm]. The ellipse angle (sway, left forefoot to right rearfoot) has a mean of − 19.34° [TR − 59.21/− 0.44°; CI − 22.52/− 16.16°] and the ellipse angle sway from right forefoot to left rearfoot has a mean of 12.75° [TR 0.09/59.09°; CI 9.00/16.33°].

**Conclusion:**

The right-to-left ratio is balanced. The forefoot-to-rearfoot ratio is approximately 1:2. Also, the body sway can be classified with 12 and 8 mm as normal. The direction of fluctuation is either approx. 19° from the left forefoot to the right rearfoot or approx. 13° the opposite. Body weight, height, and BMI were comparable to the German average of women in a similar age group, so that the measured standard values are representative and might serve as baseline for the normal function of the balance system in order to support the diagnosis of possible dysfunctions in postural control.

## Background

For an ideal regulation of the postural control, information from many different body systems such as the visual, vestibular, and proprioceptive systems must be integrated into the central nervous system [1–4]. For an undisturbed function of the postural control with the aim of maintaining balance and maintaining an upright posture, a complex network-like cooperation of the central nervous system is necessary [5–7]. If there are functional deficits or contradictory afferent information coming from the various body regions, diffuse symptoms such as vertigo, gait insecurity, or other balance disorders can occur [6, 8]. Standard values can be helpful for comparison purposes to detect deviations from a normal postural control.

However, posturographic parameters are not the same for everybody. For instance, they are different in men and women, concluding that the sex of a subject influences his or her body balance [9, 10]. Those gender-specific balance differences might be due to diverging position of the body's center of pressure [9], a different constitution of the soleus muscle [10, 11], and on a different hormonal cycle [12] of men and women.

So far, though, only a few studies have dealt specifically with the sex of the subjects [9], [13, 14], whereas other authors [15–17] have collected posturographic parameters in heterogeneous subject groups:

Syed et al. [15] investigated the body weight distribution of 628 (387m/241f, 6–89 years) subjects. They could determine a symmetrical distribution between the right and left foot and an almost symmetrical load on the forefoot and rearfoot. Using a foot pressure plate, the values 0.35–0.44 kg/sensor of the plate were measured for the right and left foot, respectively, which corresponds to a ratio of 1:1 (i.e., 50% each). For the forefoot, the value 0.71 kg/sensor and for the rearfoot, 0.81 kg/sensor was measured. This corresponds to a ratio of 1:1.13 and thus a percentage body weight distribution of 46.05% on the forefoot and 52.63% on the rear foot. As a result, all quadrants were almost equally loaded (forefoot right and left each approx. 23%, rearfoot right and left each approx. 26%).

Cavanagh et al. [16] recorded the percentage foot pressure distribution of a heterogeneous, healthy group of subjects (*n* = 107; 66m/41f) by means of a foot pressure plate with 60% on the rear foot, 8% on the metatarsus, and 28% on the forefoot.

Pomarino and Pomarino [17] determined the average percentage load on the forefoot of 267 subjects (193m/238f, 11–69 years) with 39.7% on the left and 39.6% on the right side using a pressure plate. Accordingly, a rearfoot load of 60.3% on the left side and 60.4% on the right side can be calculated. Besides Groschopp [9], who examined the position of the body’s center of gravity depending on the sex, Scharnweber et al. [14] concentrated on the evaluation of posturographic standard values for men with the age of 18–35 years and Doerry et al. [18] on standard values for women (21–30 years).

Groschopp [9] examined posturographic data of 67 male and 84 female volunteers aged 4–34 years. He found a significant correlation between age and position of the center of gravity in the sagittal plane. According to his study results, compared to women, men tend to have a more anterior center of gravity with a distance of 104.26 ± 25.27 mm to the reference line, whereas women have a significantly more posterior center of gravity (90.67 ± 22.07 mm).

Doerry et al. [18] also collected norm values for postural control of 106 healthy women aged 21 to 30 years. Average values for the load on the left and right foot were calculated at 49.91 and 50.09%, respectively, and on the forefoot and rearfoot at 33.33 and 66.67%, respectively. The body sway in the frontal plane was 9.50 mm and 13.00 mm in the sagittal plane. In a similar experimental setup, Scharnweber et al. [14] collected standard values for 87 healthy men aged 18 to 35 years. They determined average values of 11.67 mm for the frontal and 17.67 mm for the sagittal body sway.

In order to generate a large data set of standard values, the present study is part of a large-scale study: in the course of the data evaluation, standard values will be collected for subjects between the age of 20 and 60 years and also over the age of 60 years [19]. Apart from their sex, the subjects will also be divided by certain age groups to distinguish the influence of age on postural control.

Thusly, a correlation between age and position of the body’s center of gravity has already been established by Groschopp [9]. So far, Scharnweber et al. [14] and Doerry et al. [18] have recorded values for men aged 18–35 years and for women aged 21–30 years, respectively, as mentioned above. Since gender-specific standard values for women in the age group 21–30 years have already been examined [18], the present study deals with women in the age group 31–40 years. The evaluated standard values can thereafter be used as a baseline for the normal function of the balance system depending on the sex and age.

## Material and methods

### Subjects

One hundred six subjectively healthy subjects aged between 31 and 40 years (average age 35.00 ± 2.98 years) volunteered in this study. Their average size was 166.0 ± 6.0 cm and their weight 64.50 ± 13.39 kg. Their BMI was calculated to be 21.60 ± 4.65 kg/m^2^ on average.

The group of participants consisted of a variety of different occupations: beneath them were 36.79% subjects with an academic occupation (*n* = 39; doctors, school teachers, university students, etc.) and 53,83% (*n* = 56) with a training occupation (sales assistants, hairdressers, laboratory assistants Librarians, office workers, etc.) and 9.38% without a (training) occupation (*n* = 11).

As exclusion criteria applied the presence of severe pain (from value 5 on the Borg scale, “strong pain” [20]), acute or chronic diseases, injuries, or operations of the musculoskeletal system within the last 2 years. Moreover, there were neither subjects with injuries or operations in the head (especially the maxillofacial area) nor subjects with neurological diseases, rheumatism, or tinnitus.

Subjects with diagnosed physical malposition or subjects who were under physiotherapeutic or orthopedic therapy at the time of the study were also excluded. In addition, the intake of muscle relaxants was forbidden. Besides this, subjects with a painful temporomandibular dysfunction (Helkimo index [21]) were also excluded from the study.

Furthermore, an approved ethical application (ethic number: 103/16) of the Department of Medicine of the Goethe University Frankfurt am Main has been submitted to conduct the study.

### Measurement systems

#### Posturography

The pressure measuring platform GB Multisens of GeBioM (Münster/Germany) was used for the measurement of the plantar pressure [N/cm^2^] as well as the frontal and sagittal body sway [mm]. The plate measured 550 × 455 mm with a height of 4 mm. The measuring area was 390 × 390 mm, in which signals from 2304 resistance sensors were collected with a sampling rate of 100 Hz per sensor (2 sensors per cm^2^ and a sum sampling rate of 500 Hz). Each sensor detected pressure changes within an area of 8 × 8 mm by means of high-impedance amplifiers in the measuring foil. According to the manufacturer, there was a maximum error rate of only ± 5% [22].

### Examination procedure

The subjects were instructed to stand on the surface of the measuring platform without shoes within a marked circle. They were asked to adopt a natural posture, with their gaze straight ahead and the feet habitually opened hip wide. The subjects were instructed to stand as still as possible during the measurement and not to speak or turn around. The measuring system recorded the body pressure distribution and the body sway of the subjects for 30 s three times.

The following parameters were used for the statistical evaluation: (a) percentage weight distribution of the left/right forefoot (%), (b) percentage distribution of left/right rearfoot (%), (c) percentage distribution of left/right foot, (d) percentage distribution of forefoot/rearfoot (%), (e) maximum body sway in frontal plane (mm), (f) maximum body sway in sagittal plane (mm), (g) ellipse area (cm^2^), (h) ellipse width and height (cm), and (i) ellipse angle (°).

### Statistical evaluation

The statistical data was evaluated using the BiAS software program (version 11.03-07/2016, epsilon Verlag). Initially, all collected values were checked for normal distribution using the Kolmogoroff-Smirnoff-Lilliefors test. Subsequently, the mean values for the normally distributed parameters and the medians for the non-normally distributed parameters were determined. Furthermore, the corresponding 95% tolerance range and the two-sided 95% confidence interval were calculated.

## Results

Table 1 shows mean and median values, tolerance range, and confidence intervals of the plantar pressure distribution and body sway.

**Table 1:** Plantar pressure distribution and body sway (mean value or median, tolerance range, confidence interval).

Plantar pressure distribution and body sway	Mean values/medians	Tolerance range		Confidence interval	
		Lower limit	Upper limit	Left limit	Right limit
**Left forefoot (%)**	19.64	9.71	29.56	18.68	20.60
**Right forefoot (%)**	*15.00*	6.56	28.30	13.33	16.33
**Left rearfoot (%)**	31.84	16.87	46.82	30.40	33.29
**Right rearfoot (%)**	32.82	19.66	45.97	31.54	34.09
**Left foot (%)**	51.46	37.02	65.90	50.06	52.85
**Right foot (%)**	48.54	34.10	62.97	47.14	49.93
**Forefoot (%)**	*33.84*	20.68	54.73	31.67	37.33
**Rearfoot (%)**	*66.16*	45.27	79.33	62.67	68.33
**Body sway in sagittal plane (mm)**	*12.00*	5.45	23.44	11.00	14.00
**Body sway in frontal plane (mm)**	*8.17*	3.33	19.08	7.67	9.33
**Ellipse area (cm**^**2**^**)**	*0.72*	0.15	3.69	0.54	0.89
**Ellipse width (cm)**	*0.66*	0.30	1.77	0.61	0.78
**Ellipse heights (cm)**	*0.33*	0.13	0.71	0.30	0.37
**Ellipse angle (°) (all directions)**	***−*** 7.01	***−*** 53.01	39.00	***−*** 11.50	***−*** 2.59
**Ellipse angle (°) (left forefoot to right rearfoot)**	***−*** *19.34*	***−*** *59.21*	***−*** *0.44*	***−*** *22.52*	***−*** *16.16*
**Ellipse angle (°) (right forefoot to left rearfoot)**	*12.75*	*0.09*	*59.09*	*9.00*	*16.33*

Values not normally distributed are printed in italics

The average load was 19.64 (± SD = 4.98%) on the left forefoot [TR 9.71%/29.56%; CI 18.68%/20.60%] and 15.00 (± 5.77%) on the right forefoot [TR 6.56%/28.30%; CI 13.33%/16.33%]. Similarly, the mean rear foot load on the left was 31.84 ± 7.52% [TR 16.87%/46.82%; CI 30.40%/33.29%]. The mean rear foot load on the right was 32.82 ± 6.61% [TR 19.66%/45.97%; CI 31.54%/34.09%].

**Fig. 1:**
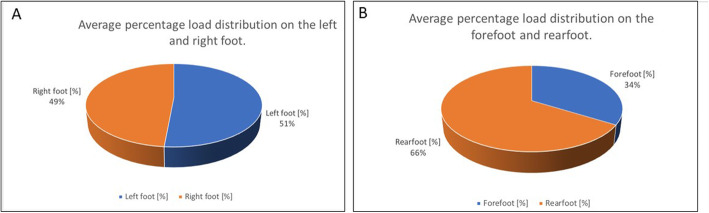
**a**, **b** Percentage body weight distribution. **a** Left foot/right foot. **b** Forefoot/rearfoot

The lateral comparison (Fig. 1a) of the left and right foot was 51.46:48.54%.

The median of the left foot was 51.46 ± 7.25% [TR 37.02%/65.90%; CI 50.06%/52.85%] and the median of the right foot was 48.54 ± 7.25%. [TR 34.10%/62.97%; CI 47.14%/49.93%]. The median forefoot load (Fig. 1b) was 33.84 ± 8.07% [TR 20.68%/54.73%; CI 31.67%/37.33%]. The rearfoot load was accordingly recorded at 66.16 ± 8.07% [TR 45.27%/79.33%; CI 62.67%/68.33%].

The median of the sagittal body sway was 12 ± 4.78 mm [TR 5.45 mm/23.44 mm; CI 11.00 mm/4.00 mm]. Values of 8.17 ± 4.03 mm were recorded for the body sway in the frontal plane [TR 3.33 mm/19.08 mm; CI 7.67 mm/9.33 mm].

The median elliptical area is 0.72 cm^2^ with a TR of 0.15 or 3.69 cm^2^ and a CI of 0.54 or 0.89. The ellipse width has a median of 0.66 cm [TR 0.30 cm/1.77 cm; CI 0.61 cm/0.78 cm] and the height of 0.33 cm [TR 0.13 cm/0.71 cm; CI 0.30 cm/0.37 cm]. The ellipse angle has a mean of − 7.01 [TR − 53.01°/39°; CI − 11.50°/− 2.59°]. The ellipse angle (sway: left forefoot to right rearfoot) has a mean of − 19.34° [TR − 59.21/− 0.44°; CI − 22.52/− 16.16°] and the ellipse angle sway from right forefoot to left rearfoot has a mean of 12.75° [TR 0.09/59.09°; CI 9.00/16.33°]

## Discussion

This study examined healthy women between 31 and 40 years of age. Their height was on average 1.66 ± 0.06 m and their weight 64.50 ± 13.39 kg. Thus, their BMI added up to 21.60 ± 4.65 kg/m^2^.

According to the results of the study of the Robert Koch Institute on Adult Health in Germany (DEGS1) [23], the average height of women in the age group 30–39 years is 1.65 m, the body weight 68.70 kg, and the BMI 25.20 kg/m^2^.

The average height of the test subjects was 1.00 cm above the evaluated average. Their mean body weight was 4.20 kg and the mean BMI 3.60 kg/m^2^ below the evaluated German average. The lower average value of the BMI can be explained by the fact that both the height and the weight of the test subjects differ from the average body measurements determined by the Robert Koch Institute. Since obesity is favored by a low socio-economic status [23], the supposedly good socio-economic situation of the subjects could be an explanation for their lower weight. Their status was rated by the ratio of university graduates and the ratio of subjects with a training occupation within the test group (in total, *n* = 95, 89.62%) [24]. Forty percent (*n* = 43) of the subjects also stated that they would exercise regularly (at least twice a week). This could be a further explanation for the lower body weight and therefore a lower BMI.

However, the body measurements of the subjects are more similar to the German average values by the Microcensus 2017 [25] of the Federal Statistical Office for the Collection of Body Measurements. According to that, the average height of women aged 30–35 (or 35–40) in Germany is 1.67 m, their weight 67 kg (or 67.90 kg), and their BMI 24.0 kg/m2 (or 24.20 kg/m^2^) [25]. Consequently, the body measurements of the subjects can be classified into those measurements and can thus be considered representative.

Taking into account the measurement error (± 5%) of the plate, the right-to-left ratio (48.54:51.46%) can be described as supposedly balanced, whereas the forefoot-to-rearfoot ratio is approximately 1:2 (33.84:66.16%). The load of the body weight is thus distributed almost evenly between the two feet, but the rearfoot is loaded considerably more than the forefoot. Of the four foot quadrants, most of the load is on the right rear foot (32.82%). The distribution of the foot load in the subjects of this study can be explained by the position of the center of gravity of the body. This is located at the level of the second sacral vertebra, slightly further in front of and below the promontory [26]. When the center of gravity is projected perpendicularly onto the support surface (center of pressure), it is located in an upright position exactly between the two feet. That means that the body’s weight load is carried equally by both feet. In the sagittal plane, the line of the perpendicular runs close to the ankle joints. Accordingly the main load of the body weight is distributed on to the rearfoot area [3, 26–28].

Syed et al. [15] were also able to determine a symmetrical distribution of body weight between the right and left foot on the basis of a symptom-free mixed group. Both, Pomarino et al. [17] and Cavanagh et al. [16] also came to the conclusion that the forefoot is much less loaded than the rearfoot. Doerry et al. [18] resulted in similar standard values for women aged 21 to 30 compared to this study (left to right ratio, 49.91:50.09%; forefoot to rearfoot ratio, 33.33:66.67%). In addition, the right rearfoot was also the most heavily loaded (34.34%). Scharnweber et al. [14] collected posturographic norm values from exclusively male subjects (18–35 years). In a lateral comparison, a slight weight tendency towards the left foot with the main load on the left rearfoot (34.67%) was found within the male subjects, whereas a tendency towards the right rear foot (32.82%) was found within the female subjects. Even though the difference is not statistically significant, that descriptive tendency in the opposite direction could be noticed.

Handiness could be a reason for the high load on the right rearfoot. Among the subjects were 102 right-handed women and four left-handed women (3.77%). For comparison, left-handed people make up 8.00% of the middle age group from 20 to 59 years of age in the European population [29]. Considering the load distribution of the left-handed women in the present study, a clear tendency towards the left side was observed, in contrast to the right-handed women. Within the left-handed subjects, the majority of the percentage foot pressure was stressed on the left foot (53.50%) and the main load was on the rear left quadrant (31.67%). It is therefore conceivable that the handiness is related to the load distribution. However, since the number of left-handed women in this study was very small (*n* = 4), this observation should be examined more closely in further analyses.

In the present study, the frontal body sway was measured 8.17 mm and the sagittal body sway 12.00 mm. These observations can be confirmed by Doerry et al. [18] measuring 9.50 mm in the frontal plane and 13.00 mm in the sagittal plane. Degani et al. [30] showed a fluctuation of 8.90 mm in frontal and 19.00 mm in sagittal direction, whereas Qiu et al. [31] found a body sway of about 17 mm in sagittal and about 7 mm in frontal direction. The amplitude of the center of pressure is clearly higher than it was measured in the subjects of the present study. A reason for that could be a lower number [30, 31] of subjects within the previous studies compared to the present one. Scharnweber et al. [14] calculated a fluctuation of 11.67 mm in frontal and 17.67 mm in sagittal plane in male subjects. Since there are differences in the physiology of men and women, those might become apparent in the body fluctuation. According to Loram et al. [11], body sway in the upright position is mainly a result of small impulsive control movements of the soleus and gastrocnemius muscles of the calf. Especially the soleus muscle acts as an antigravity muscle. According to Farenc et al. [10], structural differences of the soleus muscle (e.g., muscle thickness, length of muscle fibers) are therefore decisive for body sway (especially in the sagittal direction). Since the soleus muscle is thicker and its muscle fibers are shorter in men than they are in women, those physiologic differences may lead to a stronger body fluctuation within male subjects [10, 11]. Groschopp [9] could also explain a lower stability in men with gender-specific differences in the structure of soleus muscle.

To conclude, these female standard values are similar to Doerry et al. [18], but clearly below the male standard values (frontal 11.67 mm/sagittal 17.67 mm) of Scharnweber et al. [14] and lower than the values of the heterogenous subject groups of Degani et al. [30] and Qiu et al. [31]. Due to a variety of factors, such as impairment of proprioception or the locomotor system, postural control deteriorates with advancing age [32, 33]. Against expectations the evaluated norm values of the present study (frontal 8.17 mm/sagittal 12.00 mm) and those of Doerry et al. [18] (frontal 9.50 mm/sagittal 13.00 mm) are close to each other, although the subjects of the present study were of a more advanced age. Thus, the results can be classified into the study results of Era et al. [34]. Although they showed a deteriorating body sway with increasing age, it only becomes noticeable from the age of 60 years and onwards. Even though the values of body sway in the present study are relatively low, they can be regarded as representative due to the female sex and the relatively young age of the subjects.

Also, the body sway can be classified with 12 and 8 mm as normal, which is reflected in the values of the ellipse. The direction of fluctuation shows two different possibilities: either from the left forefoot to the right rearfoot (19°) or from the right forefoot to the left rearfoot (13°). Similar to the fluctuation, the ellipse area (72 mm^2^) is also comparable with the values of other authors (66.03 ± 29.07 mm^2^) [35]. In contrast, a study with older participants (< 65 years) found a much smaller ellipse area of 44 ± 19 mm^2^ [36]. This can be reconciled with the statement of Era et al. [34], which described a decrease of body sway from the age of 60 years.

## Conclusions

Since the body measurements of the test subjects are similar to the average values of German females, this study can be considered representative in terms of weight, height, and BMI. The percentage body pressure distribution of the subjects was almost evenly on the left and right foot. Whereas the forefoot-rear foot ration was nearly 1:2. The body sway can also be classified as normal with 12 and 8 mm, which is reflected in the values of the ellipse. Its area is comparable to that of other subjects in a similar age group. The direction of fluctuation is marginal from the left forefoot to the right rearfoot. In future, the collected standard values can be used as a baseline for the comparison with diseased patients.

## Data Availability

The datasets supporting the conclusions of this article are included within the article.

## Abbreviations

BMI: Body mass index
TR: Tolerance range
CI: Confidence interval
